# Sensitive detection of noradrenaline in human whole blood based on Au nanoparticles embedded vertically-ordered silica nanochannels modified pre-activated glassy carbon electrodes

**DOI:** 10.3389/fchem.2023.1126213

**Published:** 2023-02-15

**Authors:** Liyuan Huang, Ruobing Su, Fengna Xi

**Affiliations:** ^1^ Aerospace Center Hospital, Beijing, China; ^2^ Department of Chemistry, Zhejiang Sci-Tech University, Hangzhou, China

**Keywords:** preactivated GCE, au nanoparticles, noradrenaline, electrochemical sensor, vertically-ordered mesoporous silica-nanochannel film

## Abstract

Sensitive determination of noradrenaline (NE), the pain-related neurotransmitters and hormone, in complex whole blood is of great significance. In this work, an electrochemical sensor was simply constructed on the pre-activated glassy carbon electrode (p-GCE) modified with vertically-ordered silica nanochannels thin film bearing amine groups (NH_2_-VMSF) and *in-situ* deposited Au nanoparticles (AuNPs). The simple and green electrochemical polarization was employed to pre-activate GCE to realize the stable binding of NH_2_-VMSF on the surface of electrode without the use of any adhesive layer. NH_2_-VMSF was conveniently and rapidly grown on p-GCE by electrochemically assisted self-assembly (EASA). With amine group as the anchor sites, AuNPs were *in-situ* electrochemically deposited on the nanochannels to improve the electrochemical signals of NE. Owing to signal amplification from gold nanoparticles, the fabricated AuNPs@NH_2_-VMSF/p-GCE sensor can achieve electrochemical detection of NE ranged from 50 nM to 2 μM and from 2 μM to 50 μM with a low limit of detection (LOD) of 10 nM. The constructed sensor exhibited high selectivity and can be easily regenerated and reused. Owing to the anti-fouling ability of nanochannel array, direct electroanalysis of NE in human whole blood was also realized.

## 1 Introduction

Noradrenaline (NE) is one of the most common neurotransmitters (Emran et al., 2018). As a catecholamine (1-(3,4-dihydroxyphenyl)-2-aminoethanol), NE is secreted by the adrenal gland and released from sympathetic nerve endings to cope with stress from pain or anxiety. In addition, NE is also directly secreted into the blood as a hormone, producing various effects (increasing heart rate and blood pressure, inducing energy storage, releasing glucose and fatty acids, raising body temperature, etc.) (Khudaish and Al-Khodouri, 2020; Liu et al., 2022). The abnormality of NE is related to many diseases including pheochromocytoma, neural crest tumor, thyroid dysfunction, etc. Therefore, sensitive and rapid detection of NE in blood is of great significance.

Up to now, the detection of norepinephrine includes ion chromatography, radioimmunoassay and enzyme-linked immunosorbent assay (ELISA), chemiluminescence, fluorescence sensor, gas chromatography-mass spectrometry (GC-MS) and liquid chromatography-tandem mass spectrometry (HPLC-MS) and so on (Zhou et al., 2015; Casadio et al., 2017). Amongst, GC-MS is highly sensitive, but the derivation pretreatment requires long time and cumbersome operation. Other detection methods also often have problems such as low detection sensitivity, expensive instruments, or the need for professional operators. Electrochemical detection of electroactive substances has the advantages of rapid detection, simple instrument, easy miniaturization and integration (Yan et al., 2020a; Gong et al., 2022a; Zheng et al., 2022a; Zhou et al., 2022a; Zhou et al., 2022b; Wang et al., 2022). In addition, the selective enrichment and permeation of analytes can be realized by modifying the electrode surface (Yan et al., 2020b; Wang et al., 2021; Ma et al., 2022a). Therefore, electrochemical sensors have great potential in the rapid detection of NE.

The ability of anti-interference and anti-fouling of electrochemical sensor is crucial to expand the practical application of electrochemical analysis. When the electrochemical sensor is employed to analyze complex samples, the electrode is easy to be contaminated by large size substances (such as cells) or biological macromolecules (e.g., proteins, etc.) in the complex matrix, leading to a significant reduction in detection accuracy and reproducibility (Zhou et al., 2020; Yan et al., 2021a). Introducing an anti-interference and anti-fouling layer on the electrode surface is efficient to realize direct determination of complex samples without tedious sample pretreatment (e.g., separation). Recent studies have proven that the modification of electrode by vertically-ordered silica nanochannels film (VMSF) can achieve nanochannel-based screening at the molecular level (Ma et al., 2020; Zheng et al., 2022b). VMSF has stable nanoscale thickness (20–200 nm), ordered and highly uniform nanochannel array (pore size of 2–3 nm), high pore density (40000 μm^−2^), and large specific surface area (Qin et al., 2018; Walcarius, 2021; Gong et al., 2022b; Gong et al., 2022c). On the one hand, most biological macromolecules (e.g., proteins, DNA, polysaccharides, cells, and bacteria) are difficult to enter nanochannel with ultrasmall size. On the other hand, the hydrophilic surface of VMSF with silanol groups (Si-OH, p*K*_a_∼2) possesses obvious electrostatic screening effect, which can repel the negatively charged substances (Sun et al., 2016; Zhou et al., 2018; Gamero-Quijano et al., 2021). The charge of VMSF can also be easily changed by introducing functional groups (e.g., amino groups), demonstrating repelling towards positively charged molecules. Thus, the interference of some charged electroactive substances can be eliminated. These characteristics endow VMSF with outstanding anti-interference and anti-fouling capabilities (Yan et al., 2021b). However, silica structure of VMSF only has electrostatic adsorption towards cationic compounds or weak coordination for metal ions, (Ma et al., 2022b; Luo et al., 2022; Yang et al., 2022; Zhang et al., 2022), lacking specific responsiveness and photoelectric activity. Despite modification and functionalization of nanochannels by introducing functional groups (e.g., amino groups) (Ma et al., 2022c) or small molecular compounds (e.g., ferrocene), (Vilà and Walcarius, 2015), the incorporation of VMSF with specific nanomaterials is potential to improve the performance of VMSF-based sensors (Han et al., 2022; Zou et al., 2022).

In this work, a VMSF-based electrochemical sensor was fabricated, which can realize direct and sensitive electrochemical detection of NE in the complex sample, whole blood. Glassy carbon electrode (GCE), the most commonly used electrode, was employed as the supporting electrode and was treated by electrochemical polarization to produced pre-activated GCE (p-GCE). After NH_2_-VMSF was grown on p-GCE (NH_2_-VMSF/p-GCE), gold nanoparticles (AuNPs) were further deposited in the nanochannels of VMSF (AuNPs@NH_2_-VMSF/p-GCE). As a typical noble metal nanomaterial, AuNPs have excellent electrocatalytic activity, conductivity and large specific surface area, which can significantly improve the detection sensitivity of the sensor. The constructed electrochemical sensor has advantages of simple fabrication and easy operation. Combined with the excellent anti-fouling and anti-interference characteristics and signal amplification from gold nanoparticles, the sensor can realize direct electrochemical detection of NE in whole blood without separation pretreatment, indicating great potential for direct analysis of complex biological samples with high sensitivity and good stability.

## 2 Materials and methods

### 2.1 Chemicals and materials

Tetraethoxysilane (TEOS), cetyltrimethylammonium bromide (CTAB) (3-glycidyloxypropyl) trimethoxysilane (APTES), potassium ferricyanide (K_3_[Fe(CN)_6_]), potassium ferrocyanide (K_4_[Fe(CN)_6_]), potassium hydrogen phthalate (KHP), norepinephrine (NE), bovine serum albumin (BSA), ascorbic acid (AA), uric acid (UA), folic acid (FA) were obtained from Aladdin (Shanghai, China). L-Cystine and sodium phosphate dibasic dodecahydrate (Na_2_HPO_4_.12H_2_O) were purchased from Macklin (Shanghai, China). Hydrochloric acid was obtained from Hangzhou Shuanglin Chemical reagent (Hangzhou, China). Ethanol, calcium chloride (CaCl_2_), potassium chloride (KCl), sodium chloride (NaCl), Zinc chloride(ZnCl_2_), oleic acid (OA) were provided from Hangzhou Gaojing Fine Chemical Industry (Hangzhou, China). Whole human blood (healthy man) was provided by Hangzhou Occupational Disease Prevention and Control Institute (Hangzhou, China) for real sample analysis. Phosphate buffer solution (PBS) was prepared using Na_2_HPO_4_ and NaH_2_PO_4_ in a certain proportion. All chemicals and reagents are used directly without further purification. Ultrapure water (18.2 MΩ cm) was used throughout the work.

### 2.2 Measurements and instrumentations

Electrochemical impedance spectroscopy (EIS), cyclic voltammetry (CV) and differential pulse voltammetry (DPV) measurements were performed on an Autolab PGSTAT302N electrochemical workstation (Metrohm, Switzerland) at room temperature. A three-electrode system was used for the above electrochemical investigation with Ag/AgCl as the reference electrode (RE), platinum electrode (1 cm × 1 cm) as the counter electrode (CE), bare or modified GCE as the working electrode (WE). For DPV measurements, the step, modulation amplitude, modulation time and interval time were 0.005 V, 0.05 V, 0.05 s, and 0.2 s, respectively. The morphology of nanochannel modified electrode were characterized by scanning electron microscope (SEM equipped with X-ray energy dispersive spectroscopy-EDX, S-4800, Hitachi, Japan). Au nanomaterials obtained after chemical etching NH_2_-VMSF by NaOH (1M) was also characterized. The images were obtained at 5 kV.

### 2.3 Preparation of p-PCE

Firstly, the supporting GCE (d = 3 mm) was successively polished with 0.5 μm, 0.3 μm, and 0.05 μm alumina power. Then the electrode was thoroughly cleaned by sonication in ethanol and ultrapure water, respectively. The cleaned GCE were then treated by electrochemical polarization. Specifically, anodization was performed by applying a constant potential (+1.8 V) on GCE for 300 s. Then, cathodic polarization was performed using cyclic voltammetry scan ranged from −1.3 V to 1.25 V for 3 cycles in PBS (0.1 M, pH 5). The pre-activated electrode was denoted as p-GCE.

### 2.4 Growth of amino functionalized VMSF on p-PCE and electrodeposition of AuNPs on nanochannel array

VMSF was grown on p-GCE by electrochemically assisted self-assembly (EASA) method (Huang et al., 2022; Deng et al., 2023). Firstly, the precursor solution was prepared. After 20 mL ethanol solution and 20 mL sodium nitrate (NaNO_3_) solution was mixed, CTAB (1.585 g) and APTES (0.318 mL) was added and the pH was adjusted to 3.0 with HCl. Then, TEOS (2.732 mL) was added and the mixture was stirred at room temperature for 2.5 h. When the three-electrode system was immersed in the precursor solution, a constant current density (−0.74 mA/cm2) was applied on p-GCE for 10 s. Then, the modified GCE was quickly taken out and thoroughly washed in ultrapure water followed with aging at 80°C overnight. The as-prepared electrodes contained CTAB micelle (SM) was named as SM@NH2-VMSF/p-GCE. Then, SM@NH2-VMSF/p-GCE was put into 0.1M HCl-ethanol solution and stirred for 5 min to remove SM to obtain electrode with open nanochannel array (NH2-VMSF/p-GCE). To further electrodeposit AuNPs on nanochannel array, NH2-VMSF/p-GCE was put into chloroauric acid (HAuCl4, 0.5%) solution and deposited at 0.5 V for different time. The obtained electrode was denoted as AuNPs@NH_2_-VMSF/p-GCE.

### 2.5 Electrochemical detection of NE and real sample analysis in whole human blood

NE was electrochemical detected in PBS (0.1 M, pH = 5) electrolyte. CV or DPV method was used to record the electrochemical signals of different concentrations of NE. To investigate the application of the sensor in real sample analysis, detection of NE in human whole blood was investigated using standard addition method. Briefly, human whole blood with artificially added NE was diluted by a factor of 50 with the electrolyte. Then, NE was detected using AuNPs@NH2-VMSF/p-GCE.

## 3 Results and discussion

### 3.1 Modification of glassy carbon electrode with VMSF

**FIGURE 1:**
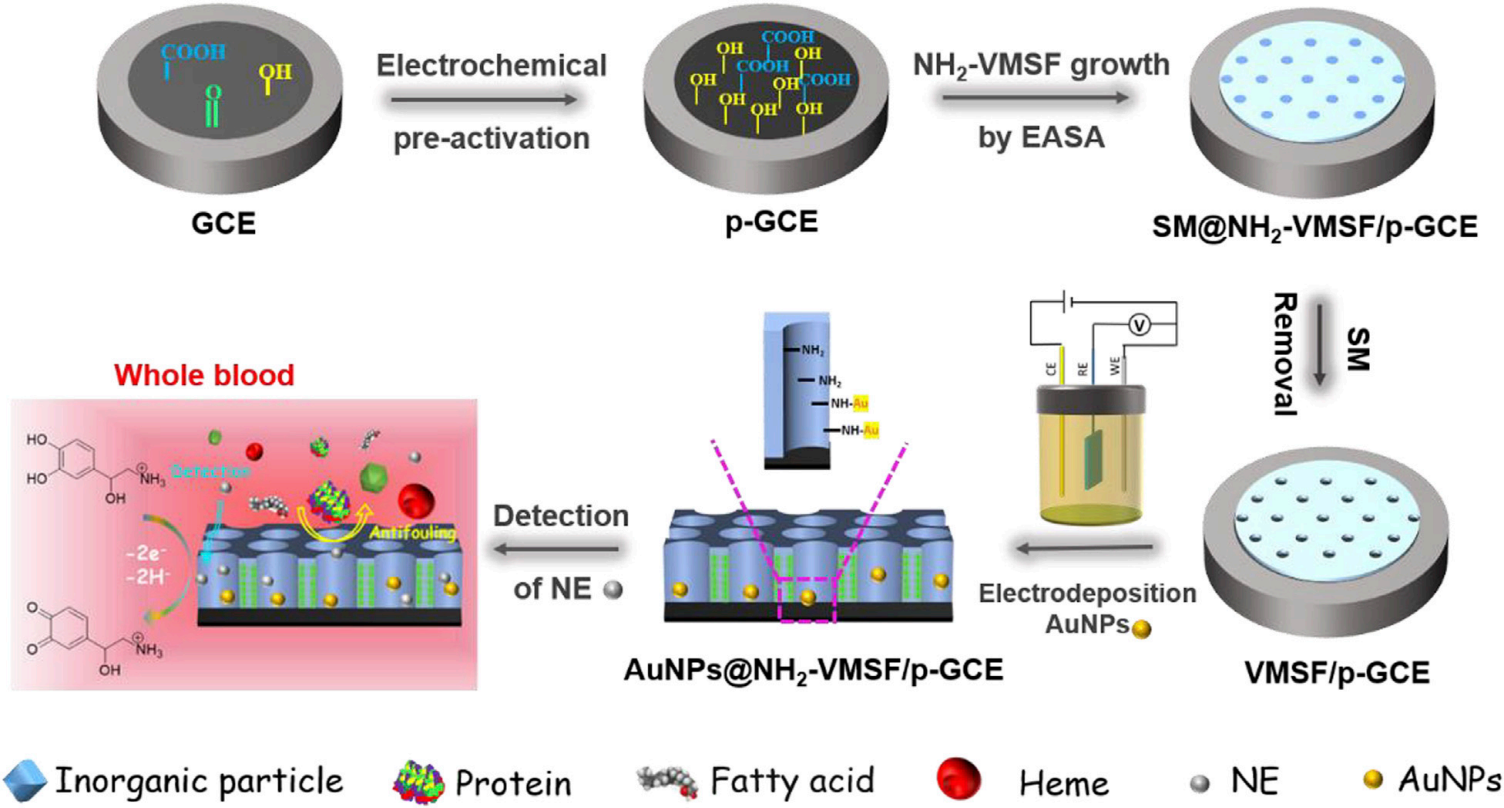
Schematic illustration for fabrication of electrochemical AuNPs/NH_2_-VMSF/p-GCE sensor and the detection of NE in complex sample.

Figure 1 illustrates the modification of VMSF on the supporting electrode and the following electrodeposition of Au nanoparticles (AuNPs) in nanochannel array. As shown, glassy carbon electrode (GCE) is selected as the underlying electrode. As known, GCE is the most widely used electrode in electrochemical sensors. As a carbonaceous electrode, GCE has unique characteristics of high chemical stability, wide potential window and good biocompatibility. However, VMSF cannot be directly and stably combined with GCE. As illustrated in Figure 1, pre-activated GCE (p-GCE) is firstly obtained through simple electrochemical polarization and stable modification of VMSF on p-GCE can be achieved without introducing an adhesive layer. This is due to the introduction of rich oxygen-containing groups on the electrode surface by electrochemical polarization. As known, electrochemical polarization is a simple and green method to prepare highly active carbon-based electrodes with no need of complex chemical reagents and tedious operations. Generally, GCE is successively anodized through electrochemical oxidation at high potential and cathodized by electrochemical reduction at low potential. Amongst, the anodizing process at high potential produces rich defects and oxygen containing functional groups through etching GCE surface. The subsequent electrochemical reduction will partially reduce the oxygen containing groups and restore the sp^2^ structure of the electrode surface, leading to the restore of the conductivity of the electrode. VMSF was then grown on p-GCE by EASA method. This method can be used to grow VMSF simply and quickly (usually 5–30 s). In the growth process, the negative charge of p-GCE resulting from the ionization of oxygen groups is beneficial to the electrostatic adsorption of cationic surfactant (CTAB) micelle (SM). At the same time, the -OH groups on p-GCE can form Si-O covalent bonds with VMSF through co-condensation reaction with Si-OH groups, thus enhancing the adhesion of the film. Due to the use of functional siloxane precursor containing amino groups, the obtained VMSF is rich in amino groups and denoted as NH_2_-VMSF. Finally, NH_2_-VMSF/p-GCE with open nano channels was obtained by removing CTAB micelles in HCl ethanol solution.

**FIGURE 2:**
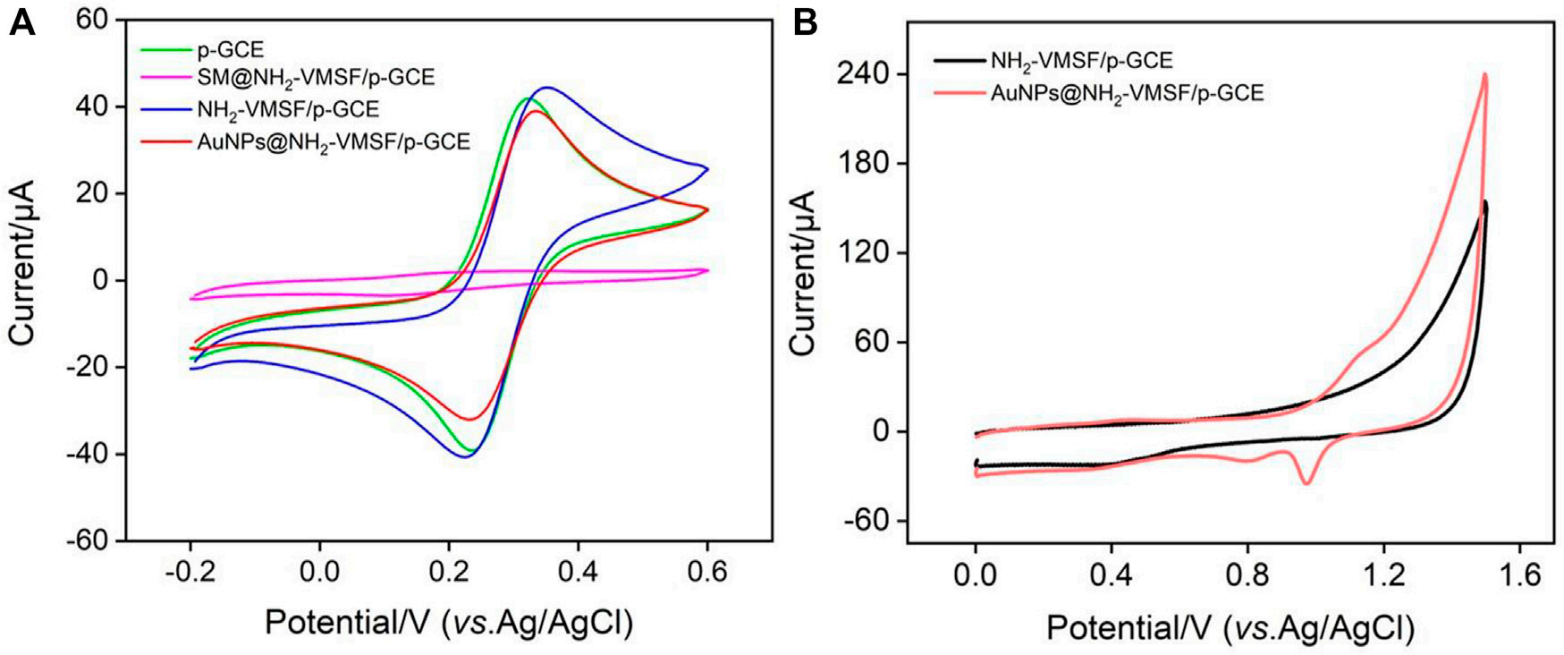
**(A)** Cyclic voltammetry (CV) curves obtained on different electrodes in KCl (0.1 M) containing Fe(CN)_6_^3^/^4-^ (2.5 mM) **(B)** CVs of NH_2_-VMSF/p-GCE and AuNPs/NH_2_-VMSF/p-GCE recorded in H_2_SO_4_ (0.1 M).

The conventional standard electrochemical probe Fe(CN)_6_^3/4-^ was used to characterize the electrochemical characteristics of each electrode in the NH_2_-VMSF modification process. Figure 2A exhibited the cyclic voltammograms (CVs) obtained on p-GCE, SM@NH_2_-VMSF/p-GCE and NH_2_-VMSF/p-GCE. As a highly active carbon electrode, p-GEC displayed remarkable redox peak of Fe(CN)_6_^3/4-^. When the nanochannel is closed by micelles, Fe(CN)_6_^3/4-^ cannot reach the surface of the underlying electrode. Thus, no obvious Faraday electrochemical signal was observed on SM@NH_2_-VMSF/p-GCE in addition to the charging current. As NH_2_-VMSF/p-GCE was rich in amino group, which had electrostatic attraction towards Fe(CN)_6_^3/4-^, the peak current of the probe on NH_2_-VMSF/p-GCE was higher than that measured on p-GCE. This phenomenon indicates that NH_2_-VMSF/ITO can promote the electrochemical response of the anion electrochemical probe.

As shown in Figure 1, gold nanoparticles (AuNPs) are *in-situ* deposited into the nano channel array by electrodeposition. It is well known that electrodeposition is a green and controllable method to prepare AuNPs with high performance. The amino groups in the nanochannels can be used as anchoring sites to increase the stability of the deposited AuNPs. The chemical composition of the modified film on electrode was characterized by X-ray photoelectron spectroscopy (XPS). As shown in Supplementary Figure S1 (Supporting information, SI), the XPS survey spectrum of NH_2_-VMSF/p-GCE contained distinct peaks corresponding to Si, C, O, and N elements resulting from the silica structure and the amine groups of VMSF. In comparison, new signal of Au_4f_ appears after the successful deposition of AuNPs on NH_2_-VMSF/p-GCE. Figure 2B was the representative CVs of NH_2_-VMSF/p-GCE and AuNPs@NH_2_-VMSF/p-GCE recorded in H_2_SO_4_ (0.1 M). As seen, no obvious redox peaks were observed on NH_2_-VMSF/p-GCE. On the contrary, peak appeared corresponding to the electrochemical oxidation of gold at the forward sweep and the reductive peak at ∼0.86 V was also observed due to the reduction of gold oxide at the reverse sweep, proving the successful deposition of AuNPs (Hou et al., 2021). Compared with NH_2_-VMSF/p-GCE, AuNPs@NH_2_-VMSF/p-GCE exhibited slightly decrease peak current of Fe(CN)_6_^3/4-^ (Figure 2A), which may be attributed to the fact that AuNPs use amino as the anchoring target, reducing the electrostatic effect of nano channels on Fe(CN)_6_^3/4-^. Supplementary Figure S2 was the top-view SEM images of NH_2_-VMSF/p-GCE and AuNPs@NH_2_-VMSF/p-GCE at different magnification. As shown, the electrode surface before and after the deposition of AuNPs was smooth, indicating that the deposition of AuNPs occurred in the nanochannels. In addition, Au nanomaterials with an average diameter of about 230 nm was observed when NH_2_-VMSF was chemically etched away from AuNPs@NH_2_-VMSF/p-GCE using NaOH (Supplementary Figure S3). The corresponding element mapping image indicated the characteristic Au signal. The large size of the Au nanomaterials mainly resulted from the aggregation of AuNPs without the spatial confinement of NH_2_-VMSF.

### 3.2 Enhanced electrochemical response of NE on NH_2_-VMSF/p-GCE

**FIGURE 3:**
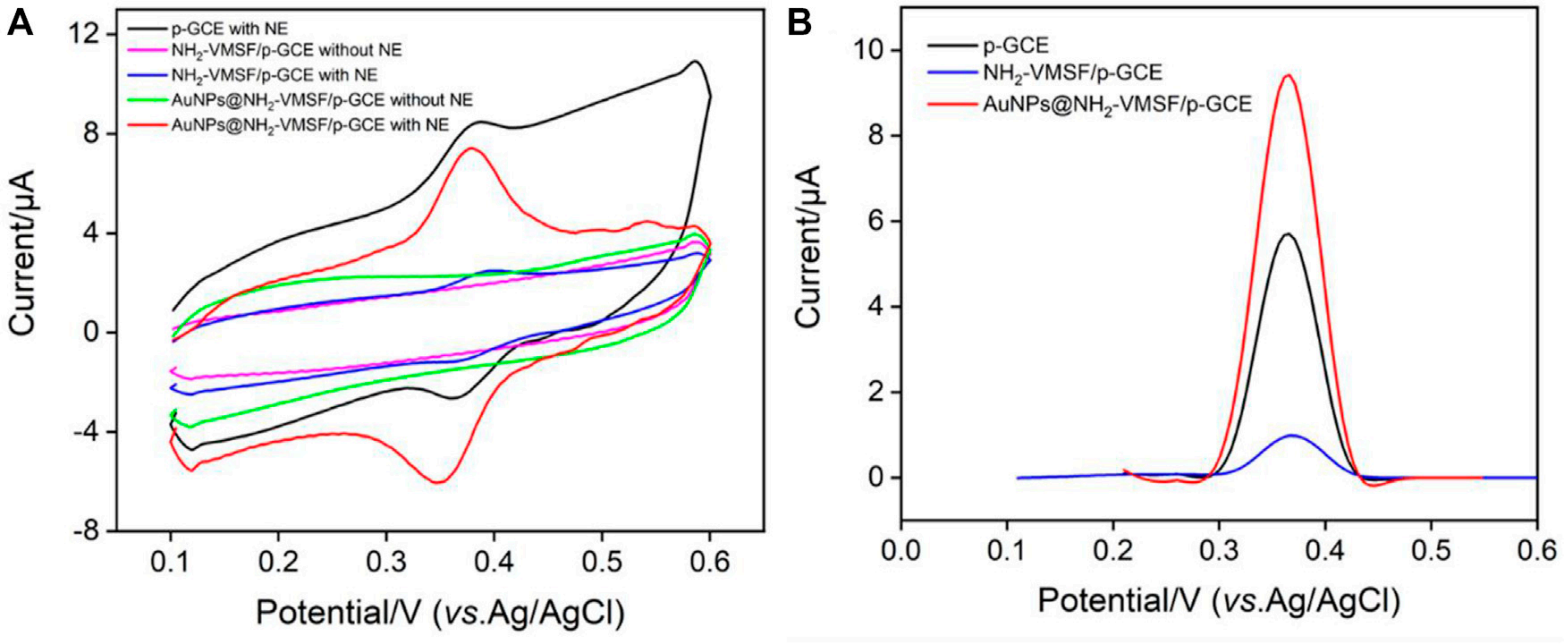
**(A)** CV curves obtained at different electrodes in PBS (0.1 M, pH = 5) without or with NE (10 μM). **(B)** DPV curves obtained at different electrodes in NE (10 μM) solution in PBS (0.1 M, pH = 5).

Figure 3 shows the comparison of CV and DPV of NE detected by GCE, NH_2_-VMSF/p-GCE and AuNPs@NH_2_-VMSF/p-GCE electrodes. Compared with NH_2_-VMSF/p-GCE, AuNPs@NH_2_-VMSF exhibited a cyclic voltammetric curve with an increased charging current, indicating the increased electroactive area after deposition of AuNPs (Figure 3A). (Ashraf et al., 2022) As seen, the redox peak of NE on NH_2_-VMSF/p-GCE was slightly lower than that obtained on p-GCE resulting from the decreased electrode area after growth of nanochannel film. On the contrary, AuNPs@NH_2_-VMSF/p-GCE showed the highest Faraday current after AuNPs were deposited into the nanochannel array (Figures 3A, B). Thus, the introduction of nanochannels and AuNPs can improve the electrochemical signal of NE. The phenomenon might be ascribed to the adsorption capacity and strong attraction of AuNPs towards NE. Compared with p-GCE, the electrochemical oxidation peak potential of NE on AuNPs@NH_2_-VMSF/p-GCE had a slight negative shift, indicating electrocatalytic ability of AuNPs.

### 3.3 Optimization of the conditions for NE detection

**FIGURE 4:**
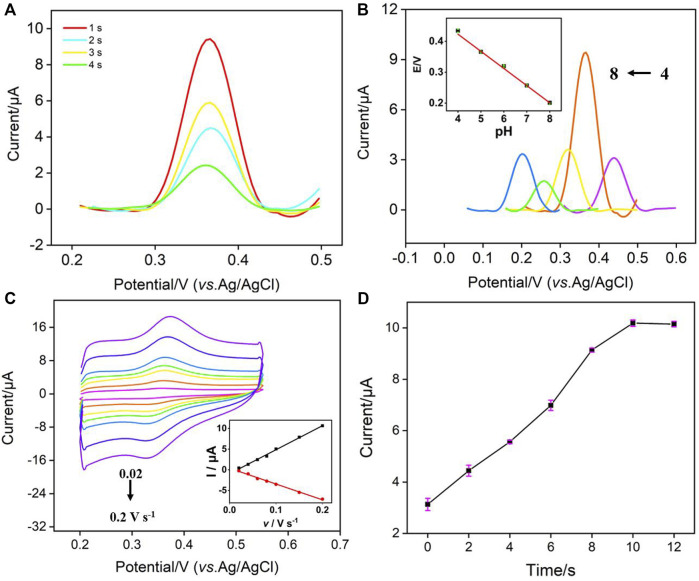
**(A)** DPV curves of NE on AuNPs@NH_2_-VMSF/p-GCE prepared using different electrodeposition time for the *in-situ* preparation of AuNPs. **(B)** DPV curves of NE (10 μM) at different pH. Inset the relationship between *E* and pH. **(C)** CV curves on AuNPs@NH_2_-VMSF/p-GCE in NE solution (10 μM) with different scan rates. Inset is the plots between peak current and scan rate. **(D)** The peak current obtained on AuNPs@NH_2_-VMSF/p-GCE at different accumulate time. The error bars represent the relative standard deviation (RSD) of three measurements.

To achieve the highest sensitivity for NE detection, the detection conditions were optimized. Figure 4A showed the effect of electrodeposition time for the formation of AuNPs on the electrochemical signal of NE. As seen, the peak current of NE decreased with the increase of electrodeposition time. Although AuNPs can improve the electrochemical signal of NE, the size of gold nanoparticles formed by too long deposition time was larger, which may hinder the diffusion of NE to the underlying electrode. Thus, the electrodeposition time was selected as 1 s in the subsequent experiment. The influence of pH on NE detection was also investigated.

Figure 4B showed the DPV curves of NE at different pH. As seen, the oxidation peak of NE moved towards the negative potential with the increase of pH (from 4 to 8), indicating that protons participate in the electrochemical oxidation and reduction process of NE. As shown in inset of Figure 4B, the oxidation peak potential (*E*) of NE had a good linear relationship with pH (*E* = −0.058 pH + 0.660, *R* ^2^ = 0.997), and the slopes were close to the slope of Nernst equation of 59 mV/pH, indicating that the number of protons involved in the electrochemical oxidation and reduction process of NE was the same as the number of electrons. Comparing the DPV oxidation peak current measured at different pH values, NE had the largest signal when pH was 5. Thus, pH 5 was selected as the optimal pH for NE detection. Figure 4C showed the CVs of NE under different scan rate (*v*). It can be seen that the peak current increased with the increase of scan rate, but the peak potential was almost unchanged, indicating high electron transfer rate. The oxidation peak current (*I*_a_) and reduction peak current (*I*_c_) were linear with the scan rate (*I*_a_ = 58.1 *v*—0.954, *R* ^2^ = 0.996; *I*_c_ = −38.2 *v* + 0.386, *R* ^2^ = 0.995), indicating that an adsorption-controlled electrochemical process of NE. Figure 4D showed the oxidation peak current of NE at different enrichment times. When the enrichment time increased, the peak current increased, and then reached a stable platform. Thus, 10 s enrichment was used in further experiments.

### 3.4 Electrochemical determination of NE

**FIGURE 5:**
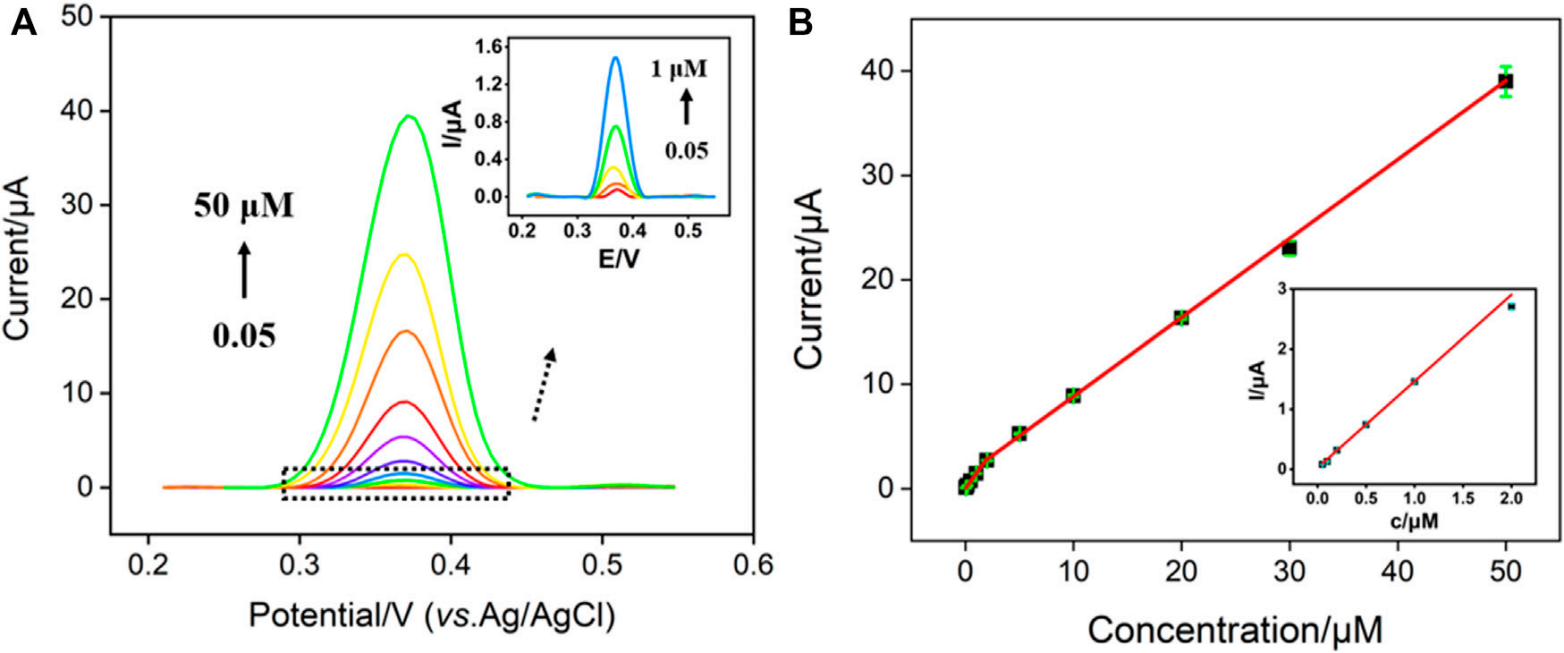
**(A)** DPV curves obtained on AuNPs@NH_2_-VMSF/p-GCE in presence of different concentrations of NE. Inset is the magnified view of the DPV curves in the low-concentration region. **(B)** The calibration curve between the peak current and the concentration of NE. Inset is the magnified view of low concentration region.

Electrochemical sensing is attractive because of advantages of low cost, fast response, easy operation, and potential for miniaturization and integration (Gao et al., 2012; Samdani et al., 2017). Under the optimized conditions, electrochemical detection of NE was investigated using DPV. Figure 5A displays the DPV curves obtained on AuNPs@NH_2_-VMSF/p-GCE in presence of different concentrations of NE. As shown, the peak current increased gradually with the increase of NE concentration. The peak current (*I*) was linearly correlated to the concentration of NE (*C*) in the range from 50 nM to 2 μM (*I* = 1.44*C* + 0.027, *R* ^2^ = 0.994) and from 2 μM to 50 μM (*I* = 0.76*C* + 1.28, *R* ^2^ = 0.998) (Figure 5B). The limit of detection (LOD) was calculated to be 10 nM using a signal-to-noise ratio of 3 (*S/N* = 3). The high sensitivity is described to the signal amplification resulting from the gold nanoparticles. Supplementary Table S1 (SI) demonstrates the comparison of determination of NE using different electrodes. As shown, the LOD is lower than that obtained from molybdenum trioxide nanowires modified GCE (MoO_3_ NWs/GCE), (Samdani et al., 2017), silica/titania material containing gold nanoparticles stabilized by chitosan modified carbon paste electrode (SiTi/AuNP/CPE) (Morawski et al., 2021), Sulphur nanodots decorated graphene oxide modified GCE (nS@GO/GCE) (Jaiswal and Tiwari, 2021), molecularly imprinted polymer (MIP) functionalized single-walled carbon nanotubes modified GCE (MIP-modified SWNTs/GCE) (Wang et al., 2017), 6-amino-4-(3,4-dihydroxyphenyl)-3-methyl-1,4-dihydropyrano[2,3-c]pyrazole-5-carbonitrile (ADPC) assisted Fe_2_O_3_@CeO_2_ coreshell nanoparticles (CNs) modified CPE (ADPC/Fe_2_O_3_@CeO_2_/GCE), (Mazloum‐Ardakani et al., 2020), MIP-coated palladium nanoparticles modified GCE (MIP-coated PdNPs/GCE) (Chen et al., 2015).

### 3.5 Detection selectivity, reproducibility, stability and the reuse performance of the fabricated sensor

**FIGURE 6:**
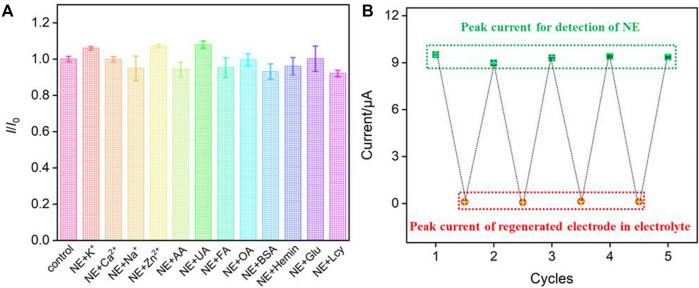
**(A)**The current ratio (*I*/*I*_0_) obtained on AuNPs@NH_2_-VMSF/p-GCE for detection of NE (5.0 μM) in the absence (*I*_0_) and presence (*I*) of 10-fold (K^+^, Ca^2+^, Na^+^, Zn^2+^, AA, UA, FA, OA, Glu, Lcy) or 10 mg/mL of other added interfering species. **(B)** The peak current obtained on original or regenerated AuNPs@NH_2_-VMSF/p-GCE in NE (10 μM) or the supporting electrolyte.

The selectivity for the detection of NE is investigated by measuring the DPV peak current in the absence (I_0_) and presence (I) of the possible interferences. Figure 6A displayed the current ratio (I/I_0_) obtained from AuNPs@NH_2_-VMSF/p-GCE for detection of NE (5.0 μM) in the absence and presence of 10-fold of K^+^, Ca^2+^, Na^+^, Zn^2+^, AA, UA, FA, OA, Glu, Lcy or 10 mg/mL of other added interfering species. When one of the above substances with a concentration of 10 times of NE was added, the current ratio on AuNPs@NH_2_-VMSF/p-GCE was close to 1.0, indicating that the peak current was basically unchanged. This shows that the sensor has high selectivity in detecting NE. To evaluate the inter-electrode reproductivity, five electrodes were fabricated in parallel. When NE (5.0 μM) was detected, a relative standard deviation (RSD) of the current value was 2.7%, indicating high reproductivity. The long-time stability of the fabricated sensors was investigated when the electroces are stored at 4°C in a refrigerator. After 7-day storage, the peak current for determination of NE (5.0 μM) remained 91% of that obtained on the first day, indicating high stability.

The fabricated AuNPs@NH_2_-VMSF/p-GCE sensor can be easily regenerated by stirring in HCl-ethanol solution for 5 min to remove the residual NE. Thus, the reuse performance for the detection of NE was also investigated. Firstly, the peak current of NE (10 μM) on AuNPs@NH_2_-VMSF/p-GCE was recorded, and then the sensor was regenerated. The electrochemical signal on the regenerated electrode was then measured in the supporting electrolyte. Then, the regenerated sensor was employed for the detection of NE again. Figure 6B showed the measured signal in NE using the original or regenerated elecrode in NE or the suppoting electrolyte. As shown, the peak current of NE on the original or regenerated AuNPs@NH_2_-VMSF/p-GCE sensor was quite similar. At the same time, the peak current obtained on the regenerated electrode in the supporting electrolyte was remarkably small, indicating that there was almost no NE residue on the electrode after regeneration. Thus, the AuNPs@NH_2_-VMSF/p-GCE sensor can be reused for the electrochemical detection of NE.

### 3.6 Real sample analysis

As known, the whole blood has complex matrix including blood cells (e.g., red cells, white cells and platelets), various electrolytes, fibrinogen, coagulation factors, proteins, etc. Commonly, direct electrochemical analysis in whole blood is challenging because of the easy fouling of the electrode. As the ultrasmall VMSF nanochannel has size exclusion effect, VMSF based electrode can exclude large sized substances such as red blood cells, showing anti-fouling effect. Therefore, VMSF modified electrode has great potential in direct electroanalysis of complex samples. In order to investigate the detection ability of the constructed AuNPs@NH_2_-VMSF/p-GCE sensor in complex samples, the standard addition method was used to investigate the NE detection performance in whole blood. Supplementary Figure S4 (SI) displayed the DPV curves obtained when different concentrations of NE was added in the diluted whole blood (by a factor of 50). Each concentration of NE was detected 3 times. As shown in Table 1, the recovery ranges from 99.9% to 102.2%, indicating high reliability in real sample analysis. In addition, the RSD for three measurement is less than 2.9%, suggesting good reproducibility during repeated detection.

**TABLE 1:** Determination of NE in human whole blood samples.

Sample	Spiked (μM)	Found, original data (n = 3, μM)	Found, average value (value ±SD, μM)	RSD (%)	Recovery (%)
Human whole blood^a^	0.50	0.503, 0.513, 0.517	0.511 ± 0.007	1.4	102.2
	5.00	4.91, 5.18, 4.95	5.01 ± 0.146	2.9	100.2
	10.00	9.92, 10.01, 10.04	9.99 ± 0.06	0.6	99.9

^a^ Samples with added NE, were diluted by a factor of 50 using the supporting electrolyte. The concentration of NE, was the value after dilution.

## 4 Conclusion

In this work, an electrochemical sensor for sensitive detection of noradrenaline (NE) was fabricated based on modification of pre-activated glassy carbon electrode (p-GCE) with vertically-ordered silica nanochannels film with rich amine groups (NH_2_-VMSF) and deposited Au nanoparticles (AuNPs). The simple and green electrochemical polarization of GCE was employed to produce p-GCE and realize stable binding of NH_2_-VMSF without adhesive layer. The electrochemically deposited AuNPs improved the electrochemical signal of NE, leading to high detection sensitivity. Sensitive electrochemical detection of NE can be achieved and the fabricated sensor exhibits good selectivity and high reuse performance. Owing to the anti-fouling ability of nanochannel, the constructed electrochemical sensor can be applied for direct detection of NE in human whole blood.

## Data Availability

The original contributions presented in the study are included in the article/Supplementary Material, further inquiries can be directed to the corresponding author.
