# Supplementary material for: Sensitive detection of noradrenaline in human whole blood based on Au nanoparticles embedded vertically-ordered silica nanochannels modified pre-activated glassy carbon electrodes
Source: Front Chem. 2023 Feb 15;11:1126213. doi: 10.3389/fchem.2023.1126213 (PMC9974660; doi:10.3389/fchem.2023.1126213)
Supplement: Supplementary file 1 [file DataSheet1.PDF]

Table S1 Comparison between determination of NE using different electrodes.

Electrode materials	*Method*	*Detection range* (μM)	*Sensitivity* *(μA μM⁻¹)*	*LOD* (nM)	*Ref.*
MoO_3 NWs/GCE	i-t	$0.1\text{-}2\times10^3$	0.0232	110	38
SiTi/AuNP/CPE	SWV	28-180	0.096	260	39
nS@GO/GCE	DPVSA	0.5-800	0.0285	260	40
MIP-modified SWNTs/GCE	CV	0.099-15	0.32	33.3	41
ADPC/Fe_2O_3@CeO_2/GCE	DPV	0.2-300	2.796	40	42
MIP-coated PdNPs /GCE	DPV	0.5-80	0.0235	100	43
AuNPs/NH_2-VMSF/p-GCE	DPV	0.05-2, 2-50	1.44	10	This work

MoO_3: Molybdenum trioxide; NWs: nanowires; GCE: glassy carbon electrode; SiTi/AuNP: silica-titania/gold nanoparticles; CPE: carbon paste electrodes; SWV: square wave voltammetry; nS@GO: sulphur nanodots grown on graphene oxide; MIP: molecularly imprinted polyme; SWNTs: single-walled carbon nanotubes; DPASV: differential pulsed anodic striping voltammetry; ADPC: 6-amino-4-(3,4-dihydroxyphenyl)-3-methyl-1,4-dihydropyrano[2,3-c],pyrazole-5-carbonitrile; Fe_2O_3 : Ferric oxide; CeO_2: cerium dioxide; DPV: Differential pulse voltammetry; MIP-coated PdNPs: molecularly imprinted polymer-coated palladium nanoparticles.

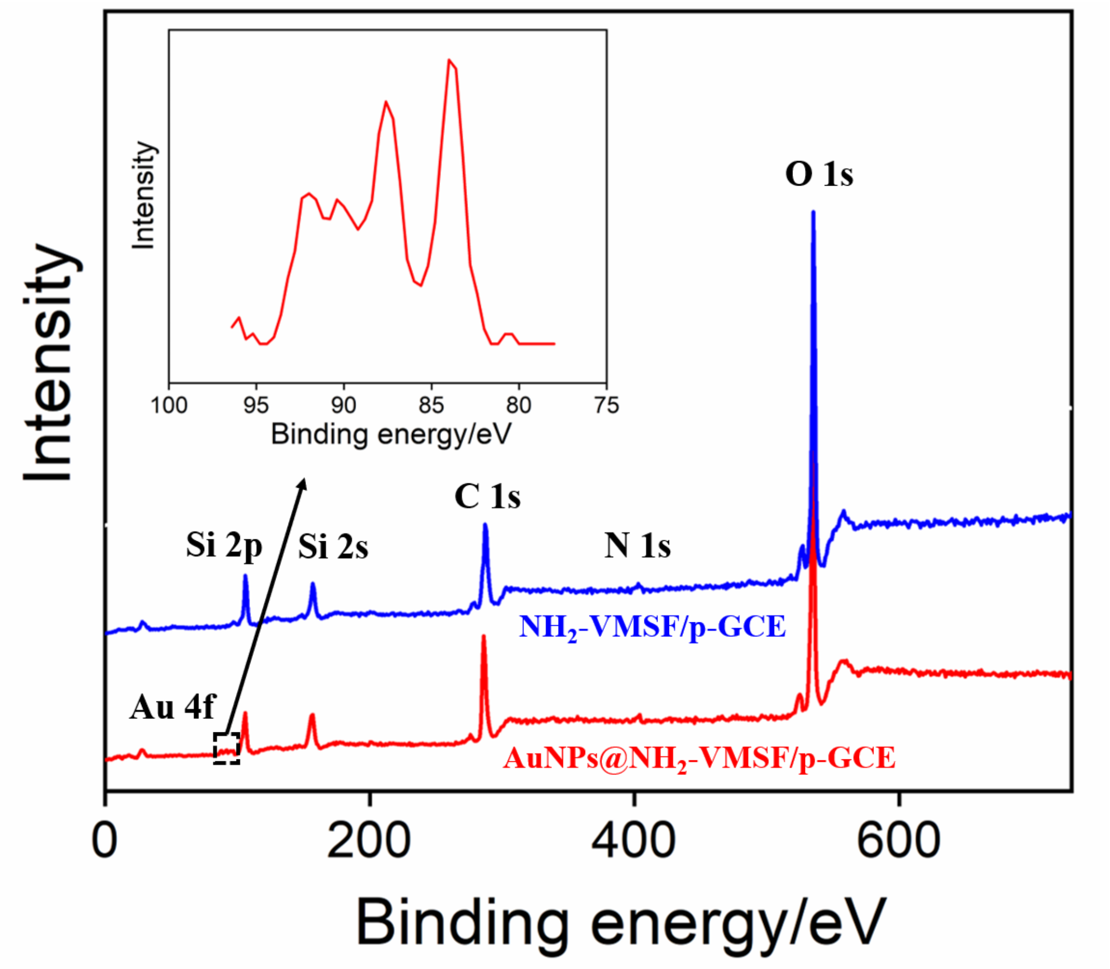

Figure S1 The XPS survey spectrum of NH_2-VMSF/p-GCE and AuNPs@NH_2-VMSF/p-GCE.

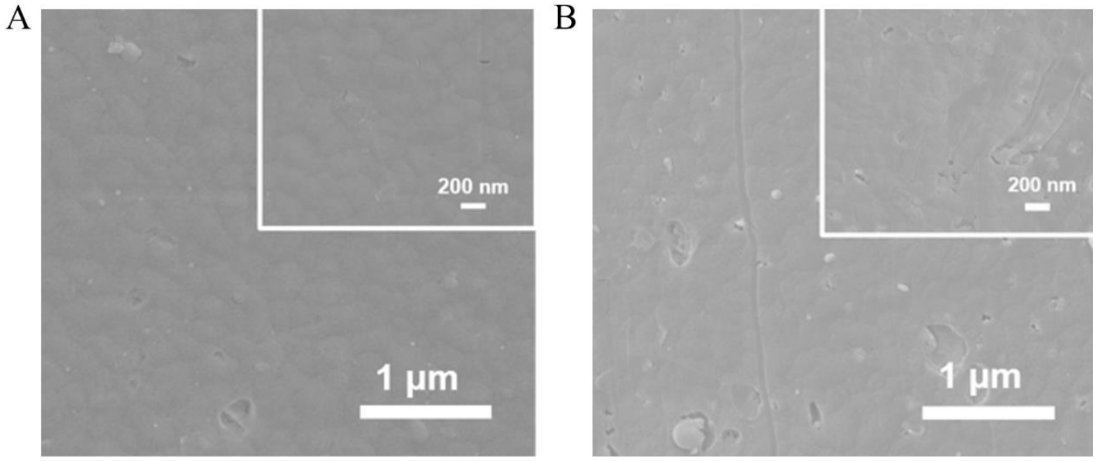

Figure S2 Top-view SEM images of NH_2-VMSF/p-GCE (A) and AuNPs@NH_2-VMSF/p-GCE (B) at different magnification.

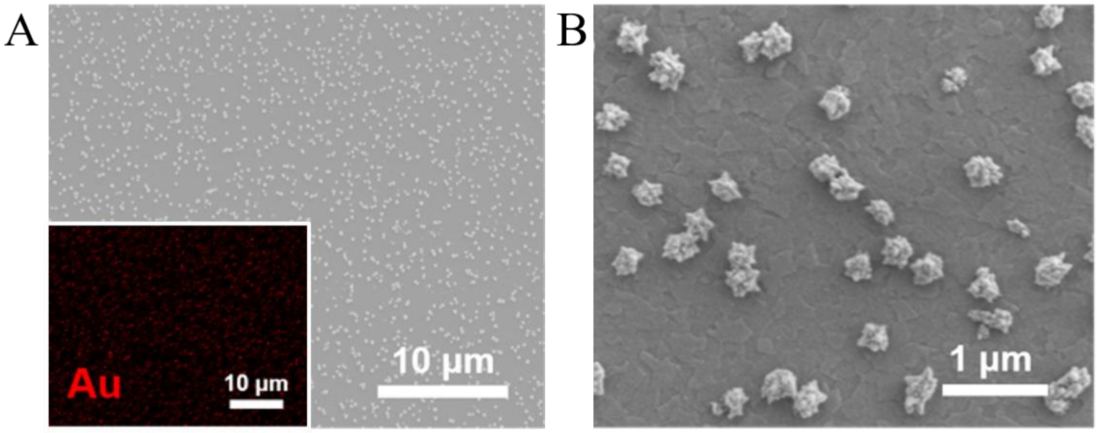

Figure S3 Top-view SEM images of Au nanomaterials after chemical etching of NH_2-VMSF in NaOH solution (1M). Inset in (A) is the corresponding EDX mapping image.

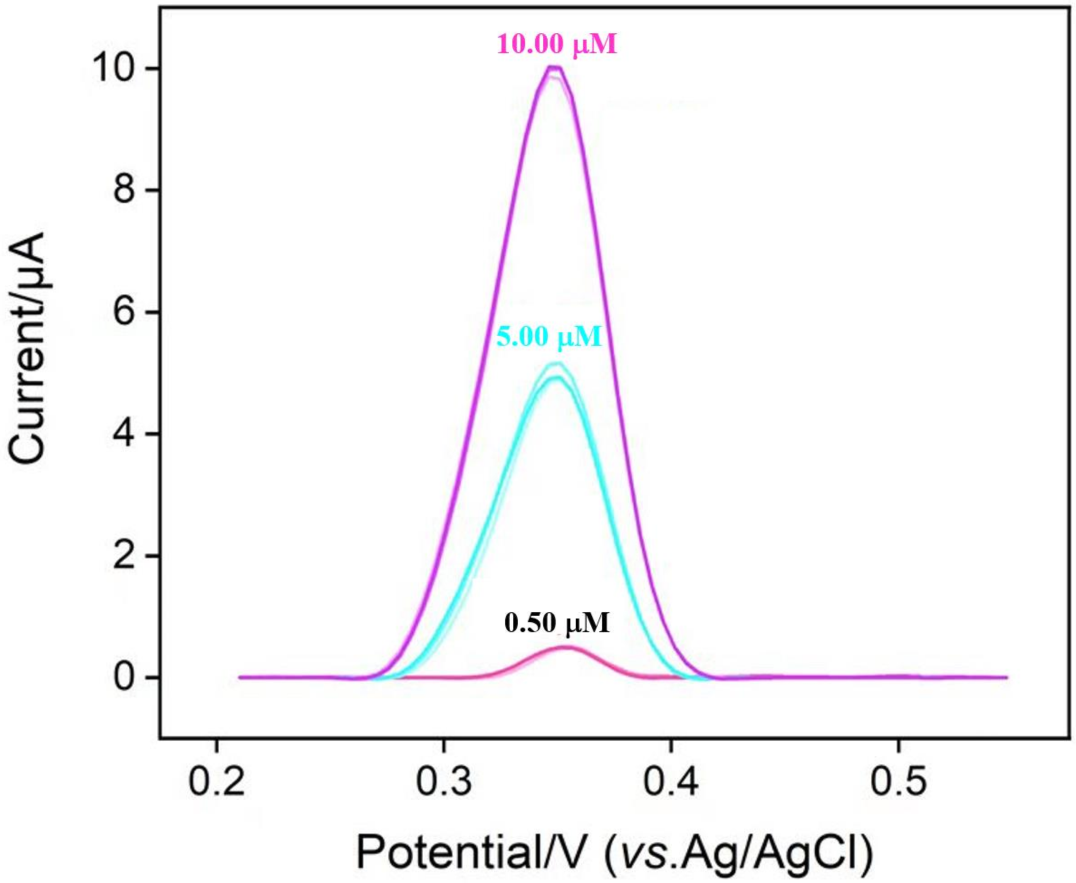

Figure S4 DPV curves obtained on AuNPs@NH_2-VMSF/p-GCE when different concentrations of NE was added in the diluted human whole blood (by a factor of 50). Each concentration of NE was detected for 3 times.
